# Modelling age-related metabolic disorders in the mouse

**DOI:** 10.1007/s00335-014-9539-6

**Published:** 2014-08-15

**Authors:** Michelle E. Goldsworthy, Paul K. Potter

**Affiliations:** Genetics of Type 2 Diabetes and Disease Model and Discovery Groups, MRC Harwell Mammalian Genetics Unit, Harwell Science and Innovation Campus, Oxfordshire, OX11 0RD UK

## Abstract

Ageing can be characterised by a general decline in cellular function, which affects whole-body homoeostasis with metabolic dysfunction—a common hallmark of ageing. The identification and characterisation of the genetic pathways involved are paramount to the understanding of how we age and the development of therapeutic strategies for combating age-related disease. Furthermore, in addition to understanding the ageing process itself, we must understand the interactions ageing has with genetic variation that results in disease phenotypes. The use of model systems such as the mouse, which has a relatively short lifespan, rapid reproduction (resulting in a large number of offspring), well-characterised biology, a fully sequenced genome, and the availability of tools for genetic manipulation is essential for such studies. Here we review the relationship between ageing and metabolism and highlight the need for modelling these processes.

## Ageing and disease

**Fig. 1:**
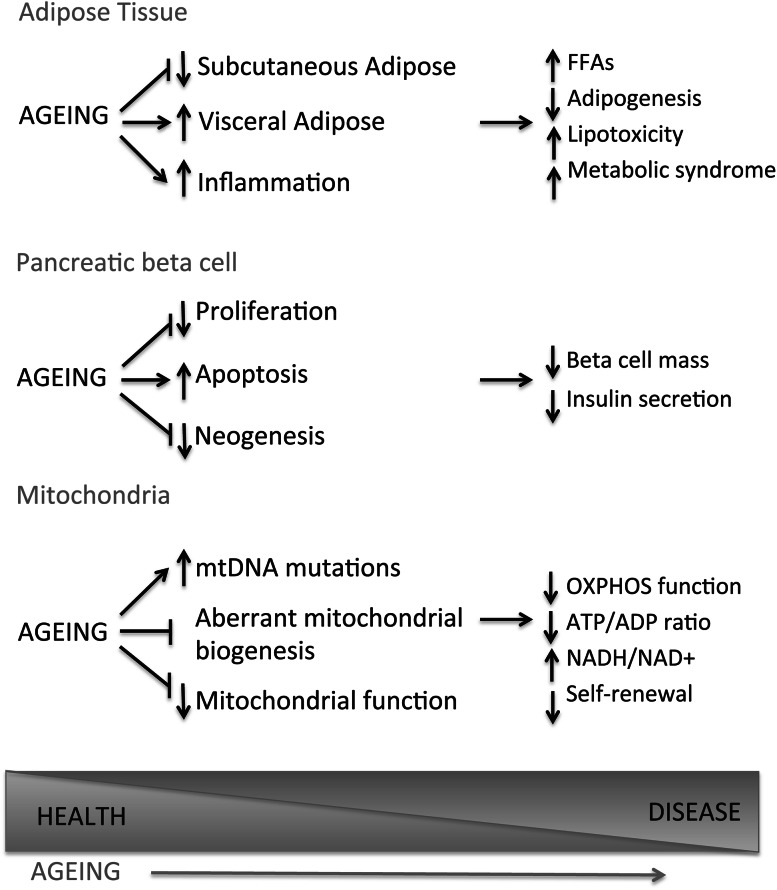
Major changes observed in important metabolic tissues due to ageing and their contributions to age-related disease

There is a clear link between age and susceptibility to a wide range of diseases [reviewed in (Niccoli and Partridge 2012)] and key among the age-related diseases that are rising in incidence is diabetes and metabolic syndrome. Ageing has specific consequences on mammalian physiology, but it also interacts with genetic variation to result in disease. There are clear genetic influences in the majority of age-related pathologies, and to fully understand disease, and how to treat it, we must understand these interactions and the influence have on the pathogenesis of these diseases (Fig. 1).

Metabolism and ageing are inextricably intertwined: Reducing calorie intake has been demonstrated in a number of studies to improve longevity, with some additional benefits to health (Robertson and Mitchell 2013; Soare et al. 2014). These nutrient-sensing pathways appear to modulate ageing as a whole and reducing calorie intake activates target of rapamycin (TOR), and involves the sirtuin family of genes (Hall et al. 2013), resulting in an increased lifespan in several model organisms (Fontana et al. 2010; Kapahi et al. 2010; Hall et al. 2013). The intimate relationship between nutrient intake and longevity is now the subject of extensive investigations to identify potential health benefits of caloric restriction. However, whilst there are clear benefits in some model organisms, the health benefits of caloric restriction are less clear-cut in primates (Mattison et al. 2012; Colman et al. 2014), and thus the general health benefits to humans are still debatable. The level of calorie intake and its influence on ageing and health highlights the close link between metabolism and ageing, and given the range of changes in metabolism with ageing (Barzilai et al. 2012). It is clear that this link needs to be understood in greater detail. The interrelationship between the ageing process(es) and metabolism is emphasised by the increased longevity in mice with a deletion of the insulin receptor (IR) substrate 1 gene (Selman et al. 2008).

A clear distinction must be made between increasing lifespan and health span; the former being an increase survival time and the latter a concept reflecting the concerns of health care professionals and researchers whose major concern is disease. Increased longevity is not a panacea against all ills and long-lived individuals still succumb to common age-related disorders (Andersen et al. 2012). The increase in lifespan that the human race has experienced over the last few decades to centuries resulted in a greatly increased disease burden and thus we still need to address pathologies on an individual basis to improve the condition of elderly patients and alleviate symptoms of these diseases.

## Insulin secretion, action and diabetes

Type 2 diabetes is the major metabolic disease associated with ageing populations, with incidence of disease typically occurring after 40 years and peaking between 60 and 74 years of age. The pathogenesis of type 2 diabetes is characterised by peripheral insulin resistance and impaired insulin secretion. Type 2 diabetes itself increases the risk for multiple other age-related diseases such as cancer, stroke, atherosclerosis, cardiovascular diseases (CVD), Parkinson’s disease, nonalcoholic fatty liver disease (NAFLD) and Alzheimer’s disease (AD). Ageing in turn can pre-dispose to diabetes and impaired glucose tolerance (IGT) through effects on insulin secretion and insulin action. Many factors contribute to this observed decrease in insulin secretion in ageing, including age-associated loss of Sirt1-mediated glucose-stimulated insulin secretion (GSIS) (Ramsey et al. 2008), decreased beta-cell sensitivity to circulating incretins (Chang and Halter 2003), and age-associated decrease in mitochondrial function and increased oxidative stress.

Defects in insulin secretion with ageing have been demonstrated in both rodents and humans. Rodent models have been utilised to demonstrate elegantly the inability of older animals to increase insulin secretion in response to increased insulin demand, which is driven by insulin resistance (van der Heide et al. 2006). Insulin secretion in the beta cell is the result of the uptake (via GLUT2) and metabolism of glucose [via Glucokinase (GCK)], resulting in the generation of ATP (mainly in the mitochondria), closure of the ATP-sensitive potassium channels leading to membrane depolarisation and influx of calcium, a rise in intra-cellular calcium levels results in exocytosis of insulin granules. The age-related changes in this process and their relevance to IGT and type 2 diabetes will be discussed briefly.

Sensing and uptake of glucose by the beta cell are the first step in insulin secretion; glucose uptake in the beta cell is facilitated by the glucose transported GLUT2 with numerous studies demonstrating GLUT2 is essential for glucose uptake. Loss of pancreatic β-cell GLUT2 expression in humans is associated with hyperglycaemia and impaired GSIS (Ohtsubo et al. 2005). Age-associated decrease in the expression of GLUT2 has been demonstrated in rodent models (Ihm et al. 2007); however, reduced GLUT2 by itself cannot explain the reduction in GSIS in islets of Goto–Kakizaki (GK) rats (Ohneda et al. 1993). After uptake, glucose undergoes oxidation resulting in the generation of ATP, which in turn is required for insulin release. The oxidation of glucose is initiated by GCK which catalyses the first and rate-limiting reaction in glycolysis. Gene expression studies have shown GCK levels were significantly increased in healthy aged rats (Frese et al. 2007) suggesting a potential mechanism to overcome age-associated glucose intolerance and insulin resistance.

Increased ATP/ADP ratio in β-cells results in depolarisation of beta-cell membrane and extracellular Ca^+^ influx into the β-cell. These changes are mediated through ATP-sensitive K_ATP_ channels and voltage-gated Ca^+^ channels. Studies in islets from 24-month-old rats compared to 3-month-old islets showed that raising the glucose concentration from 3 to 5.6 and 16.7 mM had no effect on K^+^ efflux and significantly diminished net uptake of Ca^2+^. These data suggest that the decreased insulin secretory response during old age is in part due to inadequate inhibition of K^+^ efflux and diminished net uptake of Ca^2+^ (Ammon et al. 1987). Elevation of cytosolic-free Ca^2+^ concentrations is required for the process of exocytosis of insulin which is the final step of insulin secretion. Insulin is stored in large dense core vesicles (LDCVs) or insulin granules and is released via exocytosis—a multistage process involving vesicle trafficking, docking and fusion with the plasma membrane.

Three main factors are involved with regulation of β-cell mass, proliferation and apoptosis of β-cells and islet neogenesis (differentiation of a progenitor cell or transdifferentiation of pancreatic non-β-cell to β-cell). However, β-cell mass is mainly controlled by the balance of proliferation and apoptosis. It has been shown that age correlates with decreased proliferation and enhanced sensitivity to glucose-induced β-cell apoptosis (Maedler et al. 2006). In cultured islets from young (2–3 month old) rats, increasing glucose concentrations decreased β-cell apoptosis and increased β-cell proliferation, whereas in old (7–8 month old) islets, increasing concentrations of glucose resulted in a linear increase in β-cell death and a decrease in proliferation. β-cell mass can slowly expand in adult rodents in response to increased insulin requirements or during pregnancy (Parsons et al. 1992), with β-cell proliferation and the capacity of the β-cell to regenerate declining with age in mice (Tschen et al. 2009). Young rodents respond to a high-fat diet (HFD) by increasing β-cell mass maintaining glucose homoeostasis, whereas old mice by contrast do not display increases in β-cell mass or proliferation and become diabetic. Islet neogenesis that occurs during embryonic development and in very early post-natal life can lead to β-cell mass expansion and has been shown to be important in increasing β-cell mass in the adult during periods of increased insulin demand such as obesity (Butler et al. 2003). Although the potential for β-cell replication appears to decline substantially with age, the rate of islet neogenesis is not affected by ageing in humans (Reers et al. 2009).

Insulin action is the ability of insulin to bind to its receptors, the net physiological effect of which is (1) the regulation of glucose homoeostasis through increased glucose uptake and decreased hepatic glucose production (via decreased gluconeogenesis and glycogenolysis) and (2) increase in lipid synthesis in adipocytes and the attenuation of the release of free fatty acid (FFA) from triglycerides in fat. Insulin resistance results when circulating levels of insulin are insufficient to regulate these process appropriately and it has been well documented that ageing is associated with a decline in insulin action (DeFronzo 1981; Rowe et al. 1983; Chen et al. 1988) which is thought to directly contribute to the high prevalence of IGT and type 2 diabetes with age (Enzi et al. 1986; Harris et al. 1987). Increase of visceral fat (VF) levels with ageing has been implicated in insulin resistance, CVD and type 2 diabetes. Several studies suggest that VF increases throughout the lifespan of adults (independent of race or sex) and that increase is independent of increases in body weight (Borkan et al. 1983, 1985; Enzi et al. 1986; Kotani et al. 1994). Experiments in which VF was extracted from 20-month-old Fischer 344 Brown Norway (FBN) rats prevented the progressive decrease in insulin action and delayed the onset of diabetes (Gabriely et al. 2002).

Impaired insulin action also leads to a marked increase in the rate of lipogenesis in adipose tissue and activation of hepatic gluconeogenesis in spite of already high circulating plasma glucose levels. This increased rate of lipolysis increases circulating FFA levels which exacerbates insulin resistance in the whole body. Lowering FFA levels overnight normalises insulin sensitivity in obese non-diabetic individuals and significantly improves insulin sensitivity in obese diabetic patients (Santomauro et al. 1999). High FFA levels may provide a unifying mechanism for insulin resistance in obesity, type 2 diabetes, lipodystrophy and ageing (Samuel et al. 2010).

Insulin resistance in the central nervous system (CNS) has been show to play an important role in regulating whole-body glucose metabolism. Like peripheral tissues (such as muscle, adipose tissue and liver), insulin signalling molecules such as the IR and its substrates (IRS) are universally expressed in the brain. Ablation of the IR in the CNS alone results in a more severe impairment in peripheral glucose homoeostasis that mice-lacking IR in the peripheral tissues (Koch et al. 2008). Studies have shown that intra-cerebroventricular administration of insulin was more effective at reducing food intake and body weight in 3-month-old rats than in 8- and 24-month-old rats, indicating the development of hypothalamic insulin resistance with age in Wistar rats (Garcia-San Frutos et al. 2007). An ageing-associated increase in central and peripheral insulin resistance could therefore contribute to diabetes.

## Ageing and obesity

Obesity is one of the major risk factors for diabetes and metabolic disease, and fat distribution alters during the ageing process. Obesity is defined by body mass index [BMI = weight (kg)/height (m^2^)], overweight is described as a BMI: 25–29.9 kg/m^2^ and obese as a BMI > 30 kg/m^2^ and is closely related to both percentage of body fat and total body fat (WHO 2011). Obesity leads to metabolic imbalances, reduces life span and accelerated cellular processes similar to those of ageing (Ahima 2009), such as deterioration of the structure and function of organs associated with oxidative stress, genetic instability and the disturbance of homeostatic pathways. 20 % of cancers in the US over the last 25 years have been predicted to be caused by weight gain and obesity (Calle et al. 2003). In European (Dykens and Will 2007) populations, the causal link between obesity and endometrial and oesophageal cancers has been suggested to be as high as 37 % (International Agency for Research on Cancer 2002). Worldwide obesity has nearly doubled since 1980, WHO figures show in 2008, more than 1.4 billion adults, 20 and older, were overweight. Of these over 200 million men and nearly 300 million women were obese. 65 % of the world’s population lives in countries where overweight and obesity kill more people than underweight, and more than 40 million children under the age of five were overweight in 2011. 44 % of the diabetes burden, 23 % of the ischaemic heart disease burden and between 7 and 41 % of certain cancer burdens are attributable to overweight and obesity (WHO 2011).

Body composition changes dramatically throughout life, total body weight increases until middle age (30–50 years) and slowly declines thereafter (Lutz et al. 2008), total fat mass peaks in early or late middle age (40–70 years) resulting in an increase in percentage of body fat (Kuk et al. 2009). By advanced old age (>85 years), adipose tissue is redistributed from subcutaneous to visceral depots plus ectopic sites such as muscle, liver and bone marrow with important metabolic complications. Manipulating adipose tissues abundance and distribution has profound effects on life span and age-related disease onset. Adipose tissue is not solely responsible for energy storage, it also has important immune, endocrine, homeostatic and thermal actions (Bray and Ryan 2006), with different fat deposits acting in distinct roles. Different fat depots undergo changes at differing rates with ageing, leading to fat redistribution from subcutaneous to visceral depots (Cartwright et al. 2007). Abdominal fat increases, whilst subcutaneous fat (especially from the lower body) decreases. Fat also accumulates in or around the heart, skeletal muscle and bone marrow, and increased VF is independently associated with mortality, insulin resistance, diabetes, CVD, cerebrovascular disease, heart failure, cognitive impairment and hepatic steatosis. The combination of glucose intolerance, insulin resistance, central obesity, dyslipidemia and hypertension constitutes metabolic syndrome (Morley 2004).

Loss of subcutaneous fat may contribute to reduced leptin-mediated β-oxidation of fatty acids, since leptin is produced primarily by subcutaneous fat with little production by VF (Kirkland and Dobson 1997). Reduced β-oxidation of fatty acids together with reduced capacity of subcutaneous fat to store triglycerides may contribute to increasing circulating FFAs in old age. High FFA levels lead to lipotoxicity, with cellular stress response activation, and apoptosis (Eckel et al. 2005) redistribution of excess circulating FFAs may in turn contribute to insulin resistance and therefore be a central mechanism of metabolic syndrome. In the pancreas, FFAs result in insulinopenia as they are highly toxic to pancreatic β-cells (Bays et al. 2008). FFAs also generate endothelial cell dysfunction and impair vasodilation additionally contributing to hypertension. In old age, even pre-adipocytes become susceptible to lipotoxicity, at concentrations at which pre-adipocytes from younger animals remain viable. These cells from older animals accumulate small lipid droplets characteristic of lipotoxicity and die due to apoptosis (Guo et al. 2007).

Ageing mice will lead to moderate obesity as in general mice are cage-bound, are relatively sedentary and have food ad libitum. However, common models used to study obesity are mice fed on a HFD or genetically modified mice predisposed to obesity such as the ob/ob mice (Mayer and Jones 1953). HFD generally consists of ~40 % fat content and induces rapid weight gain, and ob/ob mice become obese within a few weeks age (Wang et al. 2014). In both cases, mice are young and obese and thus missing important aspects of an ageing physiology. Recent studies have demonstrated a difference in gene expression in fat deposits between young, obese mice and old, obese mice (Liu et al. 2013).

## Mitochondrial degeneration

The primary function of mitochondria is to produce ATP through the process of oxidative phosphorylation and is unique among the cellular organelles as they contain their own genetic information mtDNA, a double-stranded circular molecule of 16.5 kb encoding 13 proteins, 22 transfer RNAs (tRNAs) and 2 ribosomal RNAs in mammals. The 13 mtDNA-encoded proteins are all components of the respiratory chain (RC) or the ATP synthase. Proper mitochondrial function is required for normal metabolism and health. Among many contributing factors to ageing is the gradual decline of mitochondrial function with age leading to progressive tissue damage via oxidative stress. Mutations in mitochondrial DNA result in a variety of phenotypes including myopathies, neuropathies, diabetes, as well as premature signs of ageing and reduced lifespan (Larsson 2010; Patti and Corvera 2010; Lee and Wei 2012). Mitochondrial dysfunction occurs at an organ-specific level in many age-related diseases including type 2 diabetes and obesity. In both rodents and humans, both obesity and diabetes are associated with reduced expression of mtDNA and reduced levels of oxidative phosphorylation in muscle, liver and adipose tissues (Larsson 2010), with caloric restriction increasing mitochondrial biogenesis and maintaining mitochondrial function (Lee and Wei 2012).

There is evidence that the number of mtDNA mutations increases with age in humans; for example, deletions in mtDNA have been observed in the aged human CNS, skeletal muscle and hepatocytes (Yen et al. 1991; Corral-Debrinski et al. 1992; Fayet et al. 2002), whereas mtDNA point mutations accumulate in ageing colonic crypts (Taylor et al. 2003). These mutations may arise as a consequence of unrepaired DNA damage, for example, damage caused by ROS or replication errors during normal mtDNA synthesis (Larsson 2010). The replication of mtDNA occurs independently of the cell cycle; therefore, a particular mtDNA molecule may be replicated many times or not at all as a cell divides (Clayton 1982). Experimental data supporting a role for mtDNA mutations in ageing were only recently obtained by the generation of mtDNA mutator mice (Trifunovic et al. 2004; Kujoth et al. 2005). The mtDNA mutator mice are homozygous for a knock-in mutation that leads to the expression of a proofreading-deficient catalytic subunit of mtDNA polymerase (PolgA^mut^), the expression of which causes excessive mtDNA mutagenesis with the formation of 3 different forms of mtDNA mutations: random point mutations, linear molecules with large deletions and molecules containing multimers of the control region.

The mtDNA mutator mice display a range of phenotypes similar to those observed with natural ageing, including kyphosis, anaemia, hair loss, alopecia, greying of the hair, hearing loss, cardiomyopathies, reduced fertility, weight loss and a shortened lifespan. Evidence strongly suggests that the high mtDNA mutation load in mtDNA mutator mice leads to the synthesis of RC subunits with amino acid substitutions that cause instability of the RC complexes (Edgar et al. 2009). mtDNA deletor mice which express a mutant-dominant version of twinkle, the replicative DNA helicase in mitochondria, accumulate low levels of large-scale mtDNA deletions. Although these mice show progressive RC dysfunction and late-onset mitochondrial myopathy, they do not display a progeroid phenotype and have a normal lifespan. This would suggest that accumulation of mtDNA deletions and progressive RC dysfunction may not be sufficient to accelerate ageing (Tyynismaa et al. 2005).

Although mitochondrial dysfunction has been shown to directly affect ageing, cellular and metabolic alterations also contribute to the ageing process by promoting secondary changes in mitochondrial energy production or mitochondrial biogenesis. Therefore, ageing phenotypes have been linked to aberrant mitochondrial biogenesis caused by impaired retrograde signalling regulated by nuclear genes and factors dependent of mitochondrial metabolism, for example, ATP, Ca^2+^, ROS, NO, NAD^+^/NADH. Insulin/IGF-1 signalling (IIS) and TOR signalling pathways are the two main nutrient-sensing pathways that have been linked to the regulation of lifespan. There is evidence that PGC-1α (Mootha et al. 2003; Anderson et al. 2008; Choi et al. 2008) and SIRT1 (Finley and Haigis 2009) are involved in the regulation of mitochondrial metabolism and lifespan. It should be noted, however, that PGC-1α does not regulate basal mitochondrial biogenesis, but is also involved in increasing mitochondrial function on demand by activating the expression of certain nuclear-encoded genes (Lee et al. 2002). There is a need for future studies to determine the relative contributions of mitochondrial dysfunction, cellular bioenergetic deficiency or changes in mitochondrial ROS production (affecting oxidative damage and signalling) to different age-related diseases. Mitochondrial dysfunction is increasingly implicated in the aetiology of drug-induced toxicities. Members of diverse drug classes undermine mitochondrial function, and routine screens need to be incorporated within the drug-development process to measure mitochondrial toxicity. Assays for mitochondrial function, cell models that better report mitochondrial impairment, and new animal models that more faithfully reflect mitochondrial dysfunction in particular ageing are required. See review Dykens and Will 2007. The Harwell metabolic ageing screen may produce potentially important models for use in drug toxicity screening.

## Inflammaging

As discussed a general consequence of ageing is obesity, although environmental factors such as diet and activity also play a significant role as well in the amassing of fat mass with age. Generally with age there is an accumulation of VF (Folsom et al. 1993) which is a risk factor for several age-related diseases. The removal of VF is beneficial to insulin resistance (Barzilai et al. 1999; Gabriely et al. 2002) and longevity (Muzumdar et al. 2008). Increased adiposity, particularly VF, has been associated with an increased production of inflammatory cytokines (Sepe et al. 2011) such as TNF-α and MCP-1. These inflammatory cytokines are thought to be produced by pre-adipocytes (Harkins et al. 2004; Chung et al. 2006) and increased macrophage infiltration of adipose tissue is observed with ageing. In addition to the increased inflammatory environment associated with VF, the senescence associated secretory profile (SASP) (Salama et al. 2014) contributes to an increased inflammatory milieu as part of the ageing physiology of mammals. The consequences of an increased systemic inflammation are far reaching as inflammation contributes to the pathophysiology of many age-related diseases such as osteoarthritis and neurodegenerative disease. Thus, the aetiology of changes in the distribution of adipose tissue during ageing and the subsequent inflammaging are important factors in age-related disease.

## Modelling age-related disorders

Model organisms have made an immeasurable contribution to our understanding of genetic contributions to disease and the resultant pathogenesis, and will continue to do so. Paramount among the model organisms has been the mouse, primarily because of the genetic tools that have been created and the homology of its biology to humans. However, the majority of mouse models are acute or early onset with significant pathology within a few months or even weeks and do not possess an aged physiology. The ageing process will impact on disease, and thus despite the clinical challenges we face being ageing population with late onset or chronic pathologies, many of the models used to study disease and in pre-clinical studies do not reflect the true nature of disease.

In order to address the challenges that health services are now facing worldwide with an ageing population, and to better understand the interactions between genetic lesions and the ageing mammalian physiology, it is necessary to place mutations in an ageing context. HFD, infection and altered light–dark samples are examples of challenges that have helped identify novel mutations resulting in disease phenotypes (Nolan et al. 2000; Bacon et al. 2004; Cook et al. 2006; Godinho et al. 2007; Georgel et al. 2008). It is clear that substantial changes occur within the mammalian physiology during the ageing process and that these can result in, or contribute to, disease (Niccoli and Partridge 2012). To develop treatments and interventions, we must understand these interactions and the only way to do this is by ageing mutant mice, using the ageing physiology as a challenge to reveal mutations that result in age-related pathologies. This is often done on an individual basis in specific disease areas such as cancer and autoimmunity but has not been attempted as part of a forward genetics screen before. In addition to age-related disease, using age-challenged mutant mice will reveal the primary and secondary effects of chronic disease over time.

The large-scale phenotype-driven screens, exemplified by ENU mutagenesis and the international knock-out mouse programmes (Acevedo-Arozena et al. 2008; Adams et al. 2013), are discovery platforms that have been eminently successful in identifying genetics lesions that result in a phenotype, thus defining novel disease-associated pathways and assigning novel functions to genes. By taking a broad-based phenotype-driven approach of mutant mice, no assumptions are made about the effects of a particular mutation and novel functions and phenotypes can therefore be associated to genes. ENU-based screens are particularly powerful because they generate point mutations, better modelling the genetic variation seen in humans, and can result in a wide variety of effects on protein function, even isolating different functional domains. Confirmation of a genetic basis of outliers is achieved by mapping of mutations, thus eliminating natural variation in phenotypes and, whilst successful, these are primarily early onset screens. We argue that to improve our modelling of age-related disease and to highlight genetic lesions that interact with an ageing physiology to result in age-related disease, it is necessary to carry out such phenotype-driven screens using ageing as a challenge. In addition to age-related disease, using age-challenged mutant mice will reveal the primary and secondary effects of chronic disease over time. Metabolism is a particularly suitable area for such studies as outlined above; there are many suitable and high throughput screens that will reveal phenotypes. Not only are primary metabolic disorders relatively easy to detect using glucose and insulin tolerance tests, fasted glucose levels and body composition screens, but also co-morbidities such as diabetic nephropathy and retinopathy can be screened for in mice using clinical chemistry analysis and visual acuity screens. Additionally, as the opportunity for exercise within standard caging conditions is limited, in general aged mice become obese. Thus, aged mice are doubly challenged by age and obesity, all too familiar condition in patients. Whilst progeroid diseases model an early ageing phenotype, these do not necessarily recapitulate the plethora of age-related pathologies.

## Metabolic and diabetic phenotyping in the ageing mouse

We are now undertaking an age-challenged phenotype-driven screen to identify genes and pathways that result in age-related pathologies using established phenotype-driven protocols (Nolan et al. 2000; Rastan et al. 2004; Acevedo-Arozena et al. 2008) with an ageing challenge. Whilst primarily focussed on mutants revealing gene function that contributes to disease, there is also the potential to identify mutations that are advantageous and protect against the consequences of ageing. Lifetime studies are confounded by expense but a detailed phenotypic pipeline may reveal mutations preventing obesity or other adverse conditions such as ageing and thus may represent mutations resulting in health benefits (Selman et al. 2008, 2009).

**Fig. 2:**
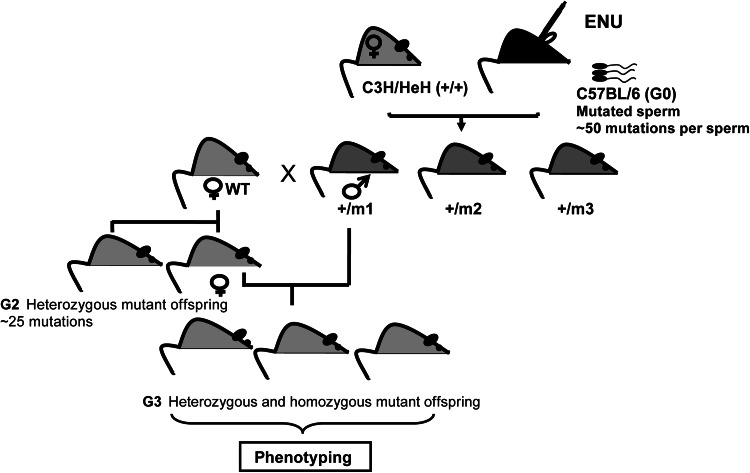
A schematic of the breeding scheme for the Harwell Ageing Mutagenesis screen. C57BL/6 J males are ENU treated and breed to C3H/HeH females to produce G1s. G1 males are further backcrossed to C3H/HeH. G2 females are mated with their G1 founder to allow the identification of recessive mutations in the G3 generation, which enter the phenotyping pipelines. Each individual G3 mouse will carry a range of ENU-induced mutations that will be homozygous, heterozygous or wild type for any given mutation. Identifying multiple mice with the same phenotype allows the identification of the causative mutation by mapping as the ENU-induced mutations will only be present in regions of the genome derived from the founder (G0) strain, in this case C57BL/6 J

In order to address the lack of suitable models of age-related metabolic disease in particular type 2 diabetes and its associated complications mice from the Harwell Ageing Screen (HAS), a G3 recessive ENU mutagenesis screen [ageing G3 pedigrees to 18 months of age (Fig. 2)] has incorporated a specific metabolism screen. Males (approximately 50/pedigree) are tested in a longitudinal study with body composition, clinical biochemistry markers and glucose tolerance followed over the lifespan of the animals. Phenodeviants within pedigrees are SNP mapped to identify candidate regions and the causative mutation identified by whole-genome generation sequencing (WGS) of the founder G1 male. Gene identification without inheritance testing is paramount to the quick identification of causative genes in an aged mouse model.

Body composition is measured with Echo MRI at 3, 6, 12 and 18 months, early time points are included to identify early onset obesity models which can be followed over the progression of disease and to identify onset of metabolic disease over the lifespan of the mouse. As expected fat mass increases with age, however, potential advantageous body composition genes can be identified in pedigrees where mice remain lean. Females in each cohort can additionally be tested if a body weight phenotype is identified within the males, to give additional statistical power. Body composition is expressed as absolute lean and fat mass in grams and can be used to identify mice with increased lean mass as well as increased fat mass. Data can also be expressed as percentage fat/lean mass to compensate for any observed differences in body size.

Fasted clinical chemistry analysis from tail bleeds is performed at 4, 7 13 and 18 (terminal) months of age. Routinely tests for fasted glucose and fructosamine (glucose homoeostasis), total cholesterol and triglycerides (lipid profile), ALT (liver function) and creatinine (kidney function) are performed at 4, 7, and 13 months, with a full biochemical screen performed on the larger terminal sample. Pedigrees with significantly elevated plasma glucose and/or fructosamine additionally undergo intra-peritoneal glucose tolerance (IPGTT) testing at 5, 8 and 14 months of age, with fasted plasma insulin levels also measured. Additional secondary phenotyping tests can be added to the screen if necessary in order to measure additional metabolic parameters, for example, whole-body oxygen consumption (Oxymax, CLAMs), food intake and activity measured and urine collected. Additionally, all mice with elevated plasma glucose enter vision screening. Additional biochemical tests can also be added to the primary screen for pedigrees with suspected renal or liver disease.

## Summary

Ageing results in a series of defined physiological effects that impact on metabolism and has direct effects on other important processes either directly or indirectly resulting in disease. To better understand the pathogenesis of disease, it is important to incorporate the ageing physiology into studies and more importantly into models of disease.
